# A Fly in the Ointment: Evaluation of Traditional Use of Plants to Repel and Kill Blowfly Larvae in Fermented Fish

**DOI:** 10.1371/journal.pone.0029521

**Published:** 2011-12-19

**Authors:** Hugo J. de Boer, Chanda Vongsombath, Jos Käfer

**Affiliations:** 1 Department of Systematic Biology, Evolutionary Biology Centre, Uppsala University, Uppsala, Sweden; 2 Department of Biology, Faculty of Science, National University of Laos, Vientiane, Lao People's Democratic Republic; 3 Université de Lyon, Université Lyon 1, CNRS, UMR5558, Laboratoire de Biométrie et Biologie Evolutive, Villeurbanne, France; AgroParisTech, France

## Abstract

**Introduction:**

In rural areas in Laos, fly larvae infestations are common in fermenting fish. Blowflies (*Chrysomya megacephala*, Diptera: Calliphoridae) are attracted to oviposit (and/or larviposit) onto fermenting fish which results in infestations with fly larvae. Knowledge of traditional use of plants to repel larvae during the production of fermented fish is common and widespread in Lao PDR.

**Research Questions:**

How effective are the most salient species in repelling, and killing fly larvae in fermenting fish?

**Material and Methods:**

The three plant species most frequently reported to repel fly larvae during an ethnobotanical survey throughout Lao PDR were tested for repellence and larvicidal activity of fly larvae infesting fermented fish. The lethality and repellence of *Tadehagi triquetrum* (L.) H. Ohashi (Fabaceae), *Uraria crinita* (L.) Desv. ex DC. (Fabaceae) and *Bambusa multiplex* (Lour.) Raeusch. ex Schult. & Schult. f. (Poaceae) were tested in an experimental design using fermenting fish in Vientiane, Lao PDR.

**Results:**

The repellent effect of fresh material of *T. triquetrum* and *U. crinita*, and the larvicidal effect of fresh *B. multiplex*, is significantly more effective than that of dried material of the same species, and the total effect (repellence and larvicidal effect combined) for each of the three species was significantly more effective for fresh than for dry material. Fresh material of *T. triquetrum*, *U. crinita*, or *B. multiplex* added on top of the fermenting fish repelled 50%, 54%, 37%, and killed 22%, 28%, and 40% of fly larvae. The total effect was not significantly different per species at 72%, 82%, and 77%, respectively.

**Discussion and Conclusions:**

The three most salient species are effective in repelling and killing fly larvae in the production of fermented fish, and may be essential to augment food safety during traditional fermentation in open jars.

## Introduction

Fermentation as a means of preservation of food is both ancient and widespread. The fermentation of fish is common among the Swedes (*surströmming*) and Norwegians (*rakfisk*) in Scandinavia, among the Yup'ik in North America (*tepa*), and widespread in Eastern Asia. The origin of fermented fish products within Eastern Asia is hypothesized to be the Mekong basin [1], where traditions and products are diverse today.

Production of fermented fish products in Eastern Asia can be separated into two main methods: a) mixtures of fish, salt and a carbohydrate source, the main varieties in Thailand known as *pla ra* and in Laos as *pa daek*; and b) mixtures of fish or shrimp and salt eaten as paste, known in Thailand and Laos as *kapi*; or fish sauce, known as *nam pa* in Laos and *nam pla* in Thailand [1], [2]. Different bacteria are responsible for different fermented fish products and stages in its production [3], with lactic-acid bacteria (e.g. *Pediococcus* spp., *Lactobacillus* spp.) playing a prominent role in lowering the pH to preserve the product. Fish-salt products are prepared by the addition of 20–30% salt to fish, and the high salt content osmotically extracts liquid from the fish tissue, thus restricting the normal microbial spoilage flora [4]. The mixtures are left to mature 1–12 months in ambient tropical temperature.

In Laos, *pa daek* is an essential ingredient of the traditional cuisine, and the product is consumed with almost every meal throughout the year. Every rural household has an earthenware jar used for the production of *pa daek*, which forms an important source of protein in the diet during the end of the dry season before the rice is harvested. *Pa daek* is made only once or twice a year, depending on the fish catch, normally at the end of the rainy season.

Sealing the mouth of the jar is a challenge in rural settings, and the main cause of spoilage is infestation with larvae of the oriental latrine blowfly, *Chrysomya megacephala* (Fabricius), and the common housefly, *Musca domestica* (Linnaeus) [5]. Both species are important vectors for the dissemination of pathogens (e.g. *Shigella* sp., *Vibrio cholerae* Pacini, *Escherichia coli* Migula, *Staphylococcus aureus* Rosenbach, *Salmonella* spp., and taenid tapeworm eggs) that cause numerous diseases in humans and animals [6]–[10].

In an earlier paper by our group [11] we reported on a large-scale survey on traditional knowledge on plant use to repel blood-sucking arthropods in Laos. Informants gave 86 species-use reports corresponding to 15 different plant species for repelling and killing fly larvae. Traditional use reports on plants used against larvae of Diptera consisted of two disparate purposes: for treatment of infested wounds in livestock, i.e., wound myiasis; and to prevent spoilage of fermented fish (*pa daek*). Most reported plant species were used to prevent spoilage of *pa daek,* and added to the mouth of the jar if infestation was observed. The most commonly reported species were *Tadehagi triquetrum* (L.) H. Ohashi (syn. *Desmodium triquetrum* (L.) DC.) (Fabaceae), *Prunus persica* (L.) Batch (Rosaceae), *Uraria crinita* (L.) Desv. ex DC. (Fabaceae) and bitter *Bambusa* spp. (*B. bambos* (L.) Voss, *B. blumeana* Schult. & Schult.f., *B. multiplex* (Lour.) Raeusch. ex Schult. & Schult. f. and *B. tulda* Roxb.) (Poaceae) [11]. Further interviews into the use of bamboo shoots as an additive to fermenting fish for killing fly larvae showed that any bitter bamboo species which would require boiling before consumption could be used. Informants reported that all four species were equally suitable for fly larvae repellence in fermenting fish production.

This study aims to test the hypothesis that traditionally used plant repellents form an effective means of repelling and killing fly larvae in fermented fish. The experiment uses a controlled test system with *pa daek* to test for biological activity against the second and third instar larvae of the oriental latrine blowfly (*C. megacephala* (Fabricius), Diptera: Calliphoridae) incubated with either of three different traditional plant repellents in three different quantities in two different states (fresh or dry). The experiment was carried out using *Tadehagi triquetrum*, *Uraria crinita*, and *Bambusa multiplex*, which are commercialized in Vientiane, Lao PDR, for fermented fish preparation.

## Methods

### Plant materials

Plant material was collected around Vientiane, and herbarium vouchers of the collections were deposited at the herbarium of the Department of Biology, National University of Laos (NUoL). *B.* multiplex shoots, 15–20×4–8 cm, were peeled, cut and longitudinally sliced into 10–20×40–50×2–4 mm slices. For dry material, the *B. multiplex* slices, and the leaves of *T. triquetrum* and *U. crinita,* were left to dry for a week away from direct sunlight. For fresh materials, the plant parts were collected, processed and used directly (within 2 hours). Plant material that was free of insect feeding marks, lesions or other damage was used. All materials were cut to small pieces (50–100 mm^2^), similar to traditional use.

### Fly larvae

Oriental latrine blowflies (*C. megacephala*) were caught using a bait trap. Five hundred grams of fresh fish heads (*Oreochromis niloticus* (Linnaeus)) were put on a tray, and covered by a tent made of netting (12×18×15 dm height-width-depth, with a 0.5 mm mesh) with an opening (10×10 cm) near the bait tray. The trap was open for three hours, 8:00–11:00 am, which was ample time for the female blowflies to locate the bait and lay eggs. After closing all flies present were identified, and the bait tray was moved to a hermetically sealed enclosure, and left for 36 hrs for egg hatching and larval development. First-instar larvae were then randomly collected from the bait, and placed in the experiment containers.

### Experimental procedure

The experimental setup consisted of 24 transparent plastic cylindrical (170h×80d mm) 850 ml jars, containing: 100 grams of 12-month old fermented fish, a mixture consisting of chunks of *Poropuntius laosensis* (Günther), *Sikukia gudgeri* (Smith), and *Mystus mysticetus* (Roberts); and 30 first-instar larvae. Each jar was covered with cloth netting (0.5 mm mesh) to prevent other flies from entering, but still allowing larvae to escape from the jar, and was placed in individual water baths to prevent disturbance by ambulatory arthropods (i.e. ants, beetles), and to be able to catch and count the escaping larvae. The baths were placed randomly in the lab. The experiment was conducted at ambient tropical temperature, in a spacious, naturally illuminated and ventilated, laboratory at NUoL. Jars were incubated with the fermented fish and fly larvae for 12 hours to allow the larvae to feed.

The experiment was carried out using the three test plant species (*T. triquetrum, U. crinita, B. multiplex*), two treatments (fresh or dried plant material), and three amounts of plant weight added (20, 30 or 40 gr), with three replicates each. The 18 control jars had no plant material added. The quantity of plant material added was chosen to be representative for traditional use. The plant material was added to the jars on top of the fermented fish after the initial 12-hour feeding incubation, and the jar was subsequently covered again with net cloth and replaced into its water bath. Each 12 hours the number of escaped larvae was recorded for each sample (Table S1). After 72 hours the jars were emptied and the total numbers of dead and surviving larvae were counted. We verified that the total number per replicate was still 30, and identified all larvae as *C. megacephala* using identification keys [12], [13] (Table S2).

Repellent activity of the plant materials was defined as larvae leaving the fermented fish after placing the test plant material and escaping the jar by creeping out through the net mesh and drowning in the water bath as measured each 12 hours during a 72-hour period. Repellence includes both direct repellence due to the plant material and deterrence after contacting or sampling the plant material. Larvicidal activity of the plant materials was defined as larvae dying in the jar after placing the test plant material as measured at the end of a 72-hour period.

### Statistics analysis

The open-source software package R was used for statistical analysis [14]. A Spearman's rank correlation test was performed on each combination of plant species (*Bambusa*, *Tadehagi*, or *Uraria*) and material state (Fresh or Dry) for quantity added (20, 30, 40 gr) for each replicate to test for a significant effect of quantity per response (escaped, dead, and escaped and dead combined). Fisher's Exact Test was used to test significance of differences in repellence, larvicidal activity and total effect between the different plant species added and the control, between dry and fresh material of the same species, between dry material of each species, and between fresh material of each species. As the six different treatments are each separately compared to the control and to one another, we need to correct the critical value of our statistical tests for multiple tests. We use a Bonferroni correction, which is known to be conservative when the statistics are dependent; six comparisons are truly independent. We thus use a critical value of 0.05/6 = 0.00833.

## Results

**Figure 1:**
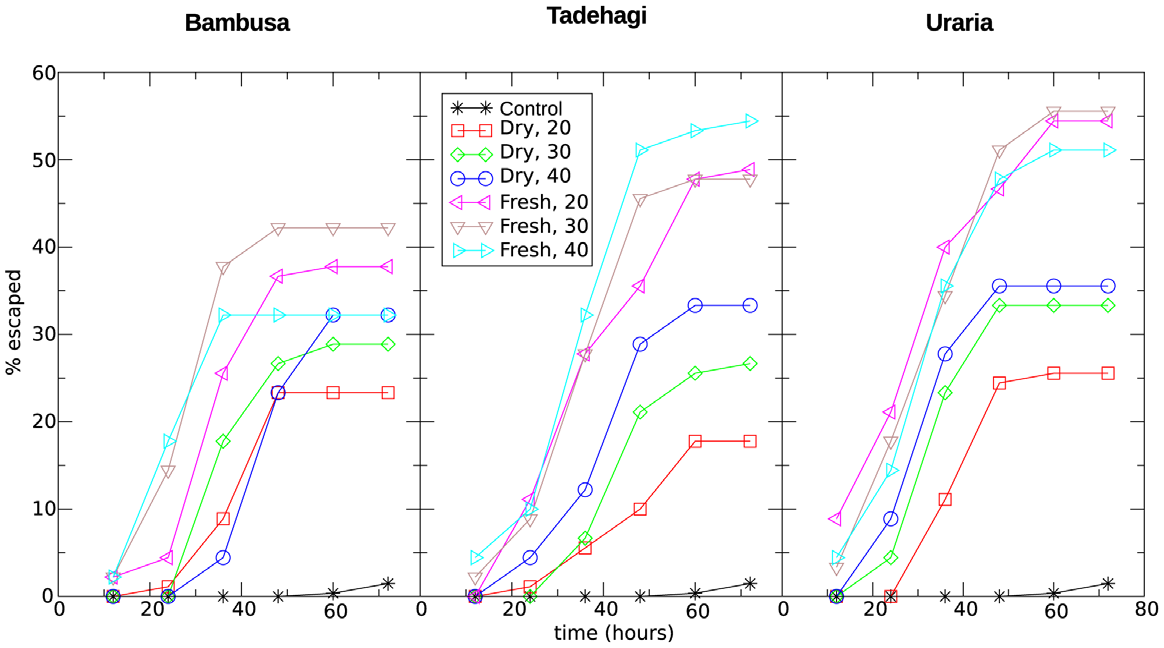
Cumulative repellence of fly larvae from fermented fish over a 72-hour period. Each point is an average percentage of three replicates, except for the control, which is averaged over 18 replicates. The control points in each graph are the same.

The number of escaped larvae from each test jar was measured every 12 hours until termination of the experiment at 72 hours and plotted in Figure 1 (for data points, see Table S1). The difference between the percentages of escaped larvae for each treatment with the controls suggests that the larvae escaped due to the repellent effect of the plants. In Figure 1, we observe that the repellent effect levels off after 48 to 60 hours. The repellent effect seems stronger for fresh than for dry plant material, while the effect of the quantity seems ambiguous.

**Figure 2:**
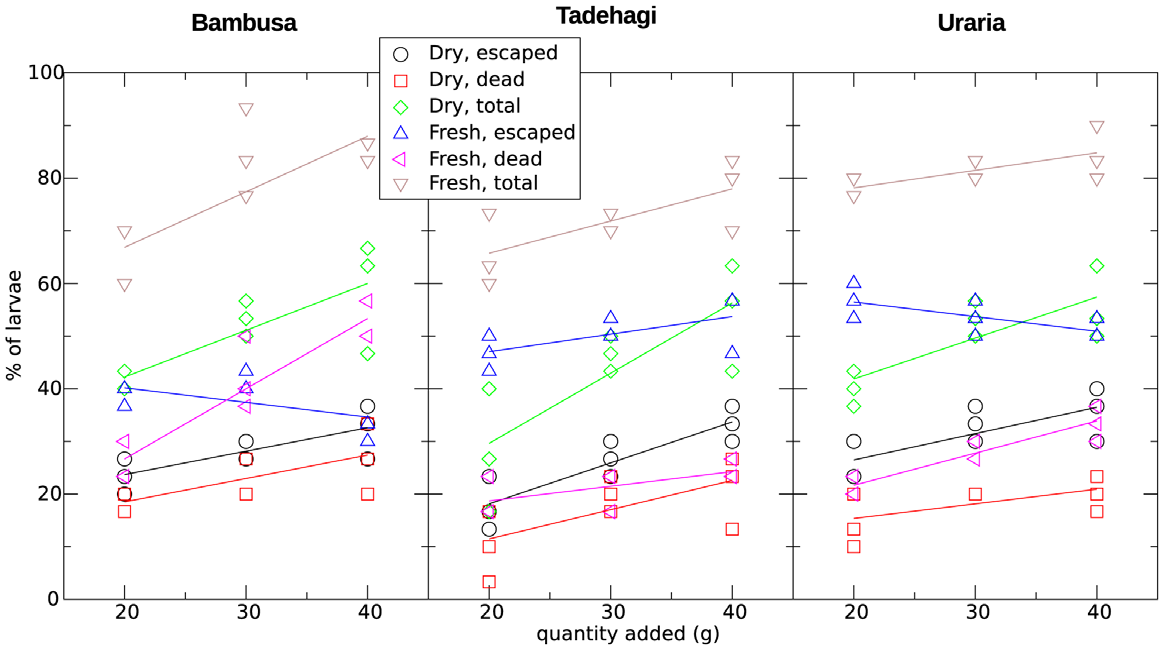
Efficacy of the treatments as a function of the quantity of added plant material. The total percentage of larvae is the sum of the percentages of escaped and dead larvae at the end of the 72-hour period. Points are shown for each replicate, and linear regression lines are drawn to guide the eye.

We thus investigate whether the quantity of plant material matters, for the repellent, the larvicidal, and the total effect. Figure 2 suggests an overall positive correlation between the quantity of material and the efficacy; The significance of Spearman's rank correlation coefficient ρ is tested and reported in Table 1. After correction for multiple testing, four significant correlations between quantity and efficacy are detected: for repellence of dry *Tadehagi*, the larvicidal activity of both fresh *Bambusa* and fresh *Uraria*, and the total effect of dry *Tadehagi*, the latter being only weakly significant (its p-value being close to the critical level 0.05/6). While there is, in a few cases, a positive effect of the amount of plant material on the efficacy, this effect is weak in the range studied here, and the quantities per treatment are taken together in the rest of the analysis.

**Table 1:** Correlation (Spearman's ρ) of the quantity of plant material added (20, 30, 40 gr) and the number of escaped and dead larvae and their sum (“Total”).

		Escape		Dead		Total	
Combination		ρ	p-value	ρ	p-value	ρ	p-value
*Bambusa*	Fresh	-0.5093	0.1614	0.9152	**0.0005**	0.7537	0.0190
*Bambusa*	Dry	0.7537	0.0190	0.7124	0.0313	0.7939	0.0106
*Tadehagi*	Fresh	0.5948	0.0911	0.6482	0.0590	0.6191	0.0754
*Tadehagi*	Dry	0.9035	**0.0008**	0.6378	0.0646	0.8203	**0.0068**
*Uraria*	Fresh	-0.6850	0.0417	0.9152	**0.0005**	0.7124	0.0313
*Uraria*	Dry	0.7300	0.0256	0.5196	0.1516	0.7441	0.0215

Spearman's Rank Correlation Test, Two-tailed, Bonferroni corrected significance p-value <0.05/6. Significant values in bold.

The effects of each treatment are summarized in Table 2; Fisher's Exact Test indicates that all treatments and effects are highly significant compared to the control (p<10^−9^). Tests to compare differences between dry and fresh material of the same species, show that repellence by *Tadehagi* and *Uraria* seems to be more effective for fresh than dry material, larvicidal activity by *Bambusa* more effective for fresh than dry material, and the total effect for all species more effective for fresh than dry material (Table 3). Significant differences in the effects between dry material of each species, and between fresh materials of each species, were found for repellence between fresh *Bambusa* and fresh *Uraria*, weakly significant between fresh *Bambusa* and fresh *Tadehagi*, and for larvicidal activity between fresh *Bambusa* and *Tadehagi*, and weakly significant between fresh *Bambusa* and fresh *Uraria* (Table 3). We conclude that there are significant differences between the effect of fresh *Bambusa* and the other species; for *Bambusa*, the repellence seems to be smaller, and the larvicidal activity larger. However, the total effects of all plant species do not differ significantly (Table 3).

**Table 2:** Repellence, larvicidal activity and total effect of each treatment (%).

Species	State	#	Escaped	Dead	Total
*Bambusa*	Fresh	9	37.4	40.0	77.4
*Bambusa*	Dry	9	28.1	23.0	51.1
*Tadehagi*	Fresh	9	50.4	21.5	71.9
*Tadehagi*	Dry	9	25.9	17.0	43.0
*Uraria*	Fresh	9	53.7	27.8	81.5
*Uraria*	Dry	9	31.5	18.1	49.6
Control		18	0.4	0.0	0.4

One-tailed Fisher's Exact Test yields p<1.0E-9 for all comparisons to the control.

**Table 3:** Comparison of plant species and state.

Repellence	*Bambusa*	*Tadehagi*	*Uraria*
*Bambusa*	0.0276	0.6282	0.4518
*Tadehagi*	**0.0032**	**6.83E-09**	0.1828
*Uraria*	**0.0002**	0.4908	**2.51E-07**
Larvicidal	*Bambusa*	*Tadehagi*	*Uraria*
*Bambusa*	**2.83E-05**	0.1063	0.2012
*Tadehagi*	**4.34E-06**	0.2299	0.8213
*Uraria*	**0.0036**	0.1098	0.0104
Total	*Bambusa*	*Tadehagi*	*Uraria*
*Bambusa*	**<1E-09**	0.0701	0.7963
*Tadehagi*	0.1661	**<1E-09**	0.1423
*Uraria*	0.2869	0.0108	**<1E-09**

Two-tailed Fisher's Exact Test. Significant values in bold. P-values for Fresh vs Fresh material on lower-left, and for Dry vs Dry material on upper-right. Center values are for within species Dry vs Fresh.

## Discussion

All three species tested in this study, *T. triquetrum*, *U. crinita*, and *B. multiplex* exhibited significant repellence and larvicidal activity against blow fly larvae. This supports the traditional use of the plant species to inhibit fly larval infestations of fermenting fish. The effects differed between species. *T. triquetrum* and *U. crinita* were more repellent than *B. multiplex*; whereas the latter had greater larvicidal activity. The total effect for all three species was similar, and no significant differences were found between the species, using either fresh or dry material. This shows that all three species were equally efficacious fermenting fish additives, although the use of *B. multiplex* is likely to lead to more dead fly larvae in the fermented fish. Dead fly larvae are washed out manually from the fermented fish before consumption. Comparison of the use of fresh or dry material shows that fresh material was significantly more effective for the total effect for all three species (p<10^−9^). This corroborates the preferred traditional use of adding fresh material on top of the fish in the fermentation jars.

### Tadehagi triquetrum

The results indicate that *T. triquetrum* has a potent repellent effect, and a moderate larvicidal effect on *Chrysomya megacephala* fly larvae. This species is used extensively in the traditional preparation of fermented products. In Laos it is widely used in the preparation of fermented fish by placing it on top of the fish in the mouth of the earthenware fermentation jar [11]. In Burma, it is reported in the traditional production of fermented fish as an effective additive in producing fly larvae-free *nga-pi*, and it is reported to have a mainly larvicidal effect [15]. In Japan, a closely related species in the subtribe Desmodiinae, *Desmodium caudatum* (Thunb.) DC. is used in the preparation of *miso* to prevent the growth of maggots [16].

The mechanism of repellent or larvicidal action is not known, but recent chemical and clinical studies suggest that prenylated isoflavonoids in *T. triquetrum* are responsible for a mild anthelminthic effect when given *in vivo* to rabbits [17], [18], and goats [19].

### Uraria crinita

The results indicate that *U. crinita*, like *T. triquetrum*, has a potent repellent effect, and a moderate larvicidal effect. In Malaysia, the crushed leaves are applied to the head as a pediculicide [20]. In North-eastern Thailand it is used in folk medicine as a vermicide and the leaves are placed on the mouth of the macerated fish jars to prevent the fish from spoiling [21]. In Laos it is widely used in the preparation of fermented fish by placing it on top of the fish in the mouth of the earthenware fermentation jar [11].

We could not find any published report on the mechanism of action of its repellent or larvicidal activity. Also, published records on secondary metabolites of *U. crinita* and its close relative *Uraria lagopodioides* (L.) DC. are sparse. Yen et al. [22] report that extracts of the root of *U. crinita* exhibits a nitric oxide-scavenging and antioxidant effects. *U. lagopodioides* is reported to have anti-inflammatory and analgesic activity [23], antimicrobial activity [24], and various new phytoconstituents have been identified to which these effects could be attributed [25]. Nevertheless a clear relation between bioactivity of secondary metabolites and the traditional use to repel fly larvae from fermenting fish is absent. Informants during the survey [11] suggested that its repellent effect was mainly due to the numerous scales on the leaves that would deter the fly larvae from crawling to the fish. However, this mechanical mode of action hypothesis does not account for the larvicidal effect of *U. crinita*, and it seems likely that so far unidentified bioactive constituents are responsible for its repellent and larvicidal effect.

### Bitter *Bambusa* spp

The four species commonly used in Laos in fermented fish preparation were reported to be *Bambusa multiplex* (Lour.) Raeusch. ex Schult. & Schult.f., *Bambusa bambos* (L.) Voss, *Bambusa tulda* Roxb., and *Bambusa blumeana* Schult. & Schult.f. Literature on the use of bamboo shoots in the preparation of fermented fish in other countries in East Asia has not been found, nor have previous studies on the efficacy of this use. The toxicity and bitterness in bamboos in caused by taxiphyllin, a cyanoglucoside with insect antifeeding properties [26]. Hydrogen cyanide is the aglycone of taxiphyllin and is released through enzymatic hydrolysis when the vacuole of the cell is disrupted. It is highly toxic to humans, and the lethal dose lies at about 50–60 mg [27]. The taxiphyllin antifeeding mechanism is found in all four common bitter bamboos, and local people reported, on the basis of their own experience, that all species were equally suitable for fly larvae repellence in fermenting fish production.

The experiment was carried out using the bitter bamboo *B. multiplex*, which exhibited a moderate repellence effect, and a strong larvicidal effect. The total effect, repellent and larvicidal effect combined, did not differ significantly between the three species.

Local informants reported that shoots of bitter bamboos had an advantage over the other repellent plant species, as the bamboo shoots would ferment together with the fish, and be suitable for human consumption after boiling when removed from the fermented fish.

### Conclusions

The traditional use of plant species as additives to fermenting fish is an effective and safe means to prevent fly larval infestations. Plant material can be found locally and procured at little or no cost, making this method both practical and cost-effective. Traditional use is currently endangered, as modern methods using salvaged plastic oil or chemical vats are employed today in parallel with the traditional use of earthenware jars. Especially in urbanized areas, the household stock of fermented fish is now sometimes made in plastic vats with sealable lids that render the use of plants obsolete. However, quantities for consumption during 1–2 weeks are still transferred to smaller earthenware jars, in which infestations are common.

## Supporting Information

Table S1 Cumulative repellence in time of fly larvae from fermented fish with traditionally used plant species.
(XLS)

Table S2 Data from traditional plants for repelling and killing blowfly larvae on fermented fish experiment.
(XLS)
